# Evaluation of STAR and Kallisto on Single Cell RNA-Seq Data Alignment

**DOI:** 10.1534/g3.120.401160

**Published:** 2020-03-27

**Authors:** Yuheng Du, Qianhui Huang, Cedric Arisdakessian, Lana X. Garmire

**Affiliations:** * Department of Biostatistics, School of Public Health, University of Michigan, Ann Arbor, MI, 48105; ‡ University of Michigan, Department of Computational Medicine and Bioinformatics, Ann Arbor, MI, 48105; † University of Hawaii at Manoa, Department of Information and Computer Science, Honolulu, HI, 96816

**Keywords:** single cell RNA-Seq, single nuclei RNA-Seq, alignment, STAR, Kallisto, Bowtie2, 10x genomics, Drop-seq, Fluidigm, accuracy

## Abstract

Alignment of scRNA-Seq data are the first and one of the most critical steps of the scRNA-Seq analysis workflow, and thus the choice of proper aligners is of paramount importance. Recently, STAR an alignment method and Kallisto a pseudoalignment method have both gained a vast amount of popularity in the single cell sequencing field. However, an unbiased third-party comparison of these two methods in scRNA-Seq is lacking. Here we conduct a systematic comparison of them on a variety of Drop-seq, Fluidigm and 10x genomics data, from the aspects of gene abundance, alignment accuracy, as well as computational speed and memory use. We observe that STAR globally produces more genes and higher gene-expression values, compared to Kallisto, as well as Bowtie2, another popular alignment method for bulk RNA-Seq. STAR also yields higher correlations of the Gini index for the genes with RNA-FISH validation results. Using 10x genomics PBMC 3K scRNA-Seq and mouse cortex single nuclei RNA-Seq data, STAR shows similar or better cell-type annotation results, by detecting a larger subset of known gene markers. However, the gain of accuracy and gene abundance of STAR alignment comes with the price of significantly slower computation time (4 folds) and more memory (7.7 folds), compared to Kallisto.

Single cell technologies allow researchers to uncover biological findings based on individual cell level. The generation of a count matrix is indispensable in order to perform downstream analyses, such as clusterin, cell type annotation (Huang *et al.* 2019 *preprint*), differential expression analysis, and pseudotime analysis on single cell RNA-seq data (Ortega *et al.* 2017); (Zhu *et al.* 2017); (Zhu *et al.* 2019 *preprint*). One of the most important and initial steps in the scRNA-seq pipeline is alignment. The goal of alignment is to find the original genomic loci of short sequence reads. The choice of aligner can directly affect the count matrix, the subsequent downstream analysis and ultimately the biological discoveries.

Recently, two methods have gained popularity in the single cell field: STAR (Dobin *et al.* 2013) and Kallisto (Bray *et al.* 2016). STAR detects the splice junctions and aligns the sequence to the reference genome non-contiguously. It can also be utilized to detect small nucleotide variations (SNVs) in scRNA-seq data (Poirion *et al.* 2018). On the other hand, Kallisto constructs k-compatibility class from the reads and performs pseudoalignment based on the de Bruijn graph. Previous benchmarking studies showed STAR was one of the most reliable reference genome based aligners in RNA-seq analysis (Baruzzo *et al.* 2017); (Engström *et al.* 2013); (Yang *et al.* 2015). Meanwhile, Kallisto was also evaluated as stable and fast for alignment-free quantification (Zhang *et al.* 2017). Both STAR and Kallisto can quantify expression, but the direct comparison between these two specific methods was lacking, especially in the scRNA-seq field.

To address this issue, we herein evaluated the performance of these two methods using real datasets obtained from different platforms (Drop-seq, Fluidigm, and 10x), some of which had orthogonal validation results of RNA FISH. We investigated the general characteristics of gene expression profiles, the accuracy of gene counts, as well as the running time and memory usage of alignment/pseudoalignment tools. We provide the first-hand reference to help researchers to decide how to perform the alignment for single cell analysis.

## Materials and Methods

### Datasets

#### Drop-seq and Fluidigm datasets:

*Dr*op-seq and demultiplexed Fluidigm RNA-seq datasets were downloaded from GSE99330 (Torre *et al.* 2018). This dataset is composed of 8640 single cells generated by Drop-seq platform and 800 single cells by Fluidigm (C1 mRNA Seq HT IFC) platforms, using WM989-A6-G3 cell line. The availability of RNA-FISH validation data on 26 genes (housekeeping genes and drug resistance markers) makes this an ideal dataset for comparing alignment methods. This dataset also is accompanied by publicly downloadable results on the same 26 genes measured by single-molecule RNA fluorescence *in situ* hybridization (smRNA FISH), a method to detect RNA within a cell using the fluorescence probe.

#### PBMC 3K scRNA-Seq datasets:

The fastq file of this dataset is downloaded from 10x genomics’ website: https://support.10xgenomics.com/single-cell-gene-expression/datasets/1.1.0/pbmc3k. The datasets were generated from a healthy donor using 10x genomics’ V1 chemistry.

#### Mouse cortex I single nuclei RNA-seq dataset:

The raw data could be obtained from Human Cell Atlas Systematic comparative analysis of single cell RNA-sequencing methods project (https://data.humancellatlas.org/explore/projects/88ec040b-8705-4f77-8f41-f81e57632f7d) (Ding *et al.* 2019 *preprint*). The sample was obtained from 1-month-old male *Mus musculus* brain cortex 1 with 10x V2 single nucleus RNA-seq methods (Flowcell lane CCJ15ANXX).

### Alignment of Drop-seq scRNA-Seq data

#### Pre-processing:

The raw Drop-seq fastq files were processed using Drop-seq (Macosko *et al.* 2015) tools version 1.13 (https://github.com/broadinstitute/Drop-seq/). Following the Drop-seq Core Computational Protocol version 1.0.1, reads with low-quality bases in the cell or molecular barcode were filtered with a default threshold setting. The adapter sequences at the 5′ and poly-A at 3′ of the reads were trimmed.

#### STAR alignment:

STAR alignment was performed for Drop-seq data using STAR version 2.5.2a provided by University of Michigan HPC. Data were aligned to reference genome GRCh38 with the corresponding annotation file from Ensemble. Default parameters were used, unless notified otherwise.

#### Kallisto pseudoalignment:

Kallisto quantification was performed for Drop-seq data using Kallisto version 0.45.1 obtained from github. Kallisto index was built with reference transcriptome GRCh38 with kmer length of 31. The suggested k-mer length of 31 can avoid ambiguities in de Bruijn graph in similar regions when k-mer length is small. Kallisto genomebam feature was used for obtaining the BAM output. Mapped reads with unknown reference genome locations were removed from BAM using Samtools version 1.9. The filtered BAM files were passed down into the Drop-seq pipeline for digital expression generation.

#### Bowtie2 alignment:

Bowtie2 version 2.3.5.1 was used (Langmead and Salzberg 2012). Bowtie2 index was generated using GRCh38 reference genome with corresponding gtf file from Ensemble.

### Alignment of fluidigm scRNA-Seq data

#### Pre-processing:

PolyA was trimmed from the 3′ end using Trim Galore version 0.4.2.

#### STAR alignment:

STAR alignment was performed for trimmed data using STAR version 2.5.2a, and aligned to reference genome GRCh38.

#### Kallisto pseudoalignment:

Kallisto quantification was performed using Kallisto version 0.45.1. Kallisto index was built with reference transcriptome GRCh38 only with the kmer length of 31. Kallisto -genomebam feature was used, and the output BAM files were filtered in the same manner as Drop-seq data.

#### Bowtie2 alignment:

the same version and index files were used, as in Drop-Seq data above.

#### Count matrix:

Aligned data were quantified using featureCounts (Liao *et al.* 2014) from Subread (Liao *et al.* 2013) version 1.6.1 with annotation for GRCh38. The configuration for featureCounts was the default: featureCounts -pPBCM–primary -T 3 -O -a /Annotation/Homo_sapiens.GRCh38.95.gtf -o aligned.bam.txt aligned.bam.

### Gini coefficient calculation and smRNA FISH validation

RNA FISH data, Drop-seq data and Fluidigm data were normalized against GAPDH, following the method described previously (Arisdakessian *et al.* 2018). Gini coefficients were calculated for 25 drug resistance markers and housekeeping genes in the same way as in SAVER(Huang *et al.* 2018).

For gene i, the level of expression across cells is sorted. Then for the sorted array of expression values, Gini index is calculated as following:

$$G_{i}=\frac{\sum_{j=1}^{n}\left(2\cdot j-n-1\right)Expression_{ij}}{n\cdot \sum_{i=1}^{n}Expression_{ij}}$$

Where j is the index for the sorted array of expression ranging from 1 to n (total number of the cell).

### Data processing and analysis on 10x Genomics PBMC 3K data

Cell Ranger filtered genes and cell matrix(available on 10x website) were processed using Seurat version 3.2.0 (Butler *et al.* 2018; Stuart *et al.* 2019), following the configuration from https://satijalab.org/seurat/v3.0/pbmc3k_tutorial.html. Clusters were annotated using provided labels by the Seurat group based on the pre-defined gene markers. Fastq files were preprocessed to fit the input scheme for both STAR and Kallisto.

#### STARsolo:

STARsolo for V1 chemistry requires two files: cell barcodes with UMIS, and cDNA read. STAR version 2.7.1a with *–solo* command was used. The STAR index was built with a read length of 98. STARsolo was configured for 14bp GemCode barcode, 10bp UMI, and 98bp transcript.

#### Kallisto bustools:

Kallisto for V1 chemistry requires three files: cell barcodes, UMIs, and cDNA reads. Kallisto version 0.45.1 was used. Kallisto bus was configured for v1 chemistry (-x 10xv1). The bus output was processed using bustools(Melsted *et al.* 2019 *preprint*) downloaded from https://github.com/BUStools/bustools. The matrix was generated following the python code available at URL: https://github.com/BUStools/BUS_notebooks_python/blob/master/dataset-notebooks/10x_hgmm_100_python/10x_hgmm_100.ipynb.

#### Kallisto With Human cDNA and intron index:

Kallisto version 0.46.1 and bustools version 0.39.3 were used. The cDNA^+^intron index and relevant files were downloaded from the github page: https://www.kallistobus.tools/velocity_tutorial.html. The pseudoalignment and sequential correction and counting processes were done following the instruction from https://www.kallistobus.tools/velocity_tutorial.html. The spliced and unspliced matrices were processed following the instruction from https://github.com/BUStools/getting_started/blob/master/velocity_tutorial.ipynb. The cells with less than 3 expressed genes, and genes expressed in less than 200 cells were removed.

#### Downstream clustering analysis:

Seurat version 3.2.0 was used for downstream analysis. The filtration criteria include min.cells = 3, min features = 200. Data were then log normalized with a scale factor of 10000 in Seurat. The cell types were annotated manually based on the FeaturePlot of each marker gene.

### Data processing and analysis on 10x mouse cortex 1 single nuclei RNA-seq data

#### STARsolo:

STAR version 2.7.3a with *–solo* command was used. For single nuclei RNA-seq data, command “–soloFeatures Gene SJ GeneFull” was used for generating counts for both exonic RNA and pre-mRNA. The STAR index was built with a read length of 50. STARsolo was configured for 16bp GemCode barcode, 10bp UMI, and 50bp transcript.

#### Kallisto with mouse cDNA and intron index:

Kallisto version 0.46.1 and bustools version 0.39.3 was used. The mouse ensembl 86 cDNA^+^intron index and relevant files were downloaded from the github page: https://github.com/pachterlab/MBGBLHGP_2019/releases. The pseudoalignment and sequential correction and counting processes were done similarly as the PBMC 3k data. The spliced and unspliced matrices were processed in Python 3.7. The cells with less than 3 expressed genes, and genes expressed in less than 200 cells were removed.

#### Downstream clustering analysis:

Seurat version 3.2.0 was used for downstream analysis. The filtration criteria include min.cells = 3, min features = 200. Data were then log normalized with a scale factor of 10000 in Seurat. The cell types were annotated manually based on the FeaturePlot of each marker gene.

### Time and memory measurement

Time and memory usages on Fluidigm dataset were measured on a dedicated group computer server cluster (consisted of 4 nodes (Dell PowerEdge C6420) of 2 X Intel(R) Xeon(R) Gold 6154 CPU @ 3.00GHz, 192GB RAM, one node (Dell Poweredge R740) with 2 X Xeon(R) Gold 6148 CPU @ 2.40GHz, 192 GB RAM, and two 16GB Nvidia V100 GPUs.) with Slurm job scheduler. One processor and 60GB memory were reserved for each job. “Resources_used.cput” and “resources_used.walltime” were collected from the job log for analysis.

### Code availability

All code for the tools configurations and analysis could be found on GitHub page: https://github.com/yhdu36/aligner

### Data availability

The authors state that all data necessary for confirming the conclusions presented in the article are represented fully within the article, figures and tables. Supplemental material available at figshare: https://doi.org/10.25387/g3.11866281.

## Results and Discussion

### Comparisons of STAR *vs.* Kallisto alignment results on Drop-Seq and Fluidigm data

STAR and Kallisto are based on different concepts. STAR is a conventional aligner that aligns to the reference genome, whereas Kallisto uses transcriptome quantification for pseudoalignment. To compare these two methods, we downloaded the raw sequencing reads from a previously published GEO data set (GSE99330) (Torre *et al.* 2018). Briefly, this dataset is composed of 8640 single cells generated by Drop-seq platform and 800 single cells generated from Fluidigm (C1 mRNA Seq HT IFC) platforms, using WM989-A6-G3 cell line as the biological material. The RNA-FISH validation data on 26 genes serve as the standard that could help validate the expression level as the result of different alignment methods. We used GRCh38 as the reference genome for STAR and GRCh38 as the reference transcriptome for Kallisto, per recommendation of the authors.

**Figure 1:**
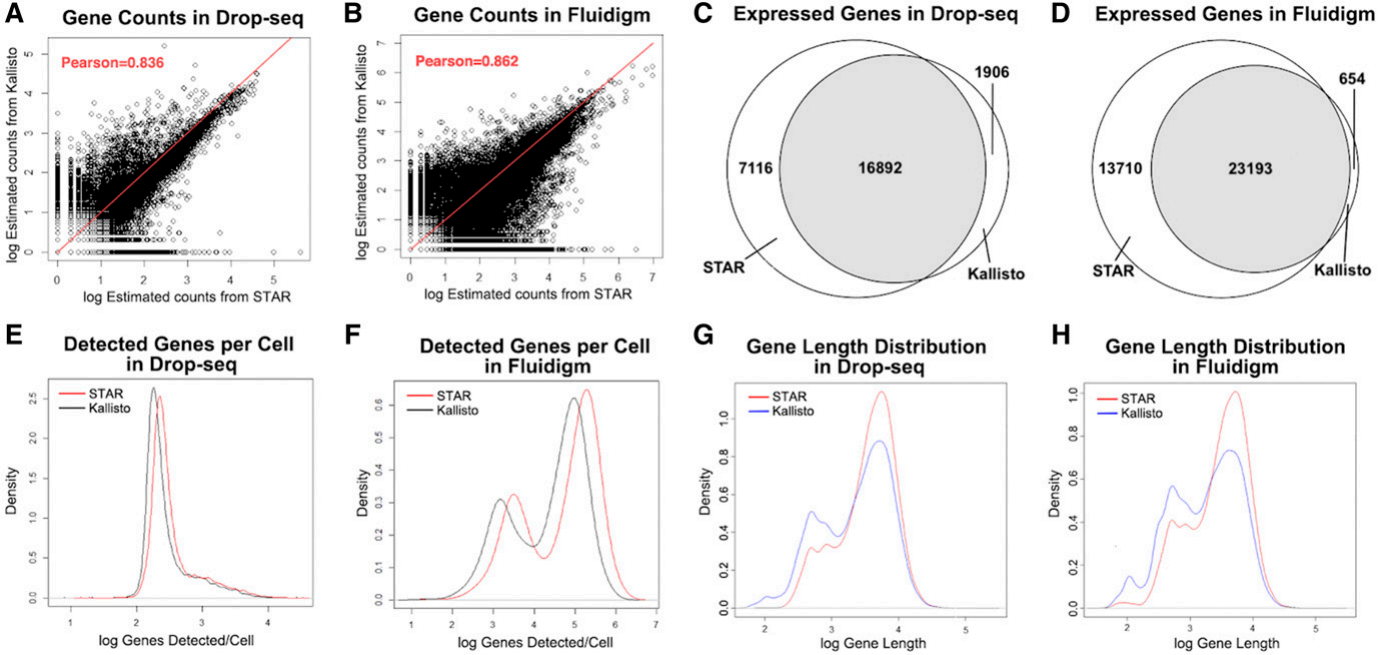
Comparison of single-cell gene expression from STAR and Kallisto Alignment. (A-B) Log 10-transformed (gene counts +1) obtained after alignment/pseudoalignment. The gene counts were calculated by taking log 10-transform on the summation of the estimated counts across all samples. The x-axis represents the expression level with STAR protocol, and the y-axis represents the expression level with Kallisto protocol. Pearson correlation coefficients are shown on the graph as “Pearson”. (C-D)Venn diagrams for the counts of expressed genes detected in Drop-seq and Fluidigm datasets. The intersect regions are the genes that are detected in common by STAR and Kallisto. (E-F) Density plots of the total number of detected genes within each cell for Drop-seq and Fluidigm data. The x-axis represents the log 10-transformed total estimated gene counts in each sample, and the y-axis represents the density. (G-H) Density plots of the length of expressed genes in Drop-seq and Fluidigm datasets. The x-axis represents the log-transformed gene length of expressed genes, and the y-axis represents the density.

For the scRNA-seq reads from Drop-seq platform, STAR has 62.40% alignment rate, compared to 35.11% pseudoalignment rate from Kallisto; for the reads from Fluidigm platform, STAR has 66.57% alignment rate, compared to 34.03% from Kallisto (Table S1). To generate the count matrix, we used STAR and Kallisto genombam command (Yi *et al.* 2018 *preprint*) followed by featureCount. Kallisto genombam command projects the pseudoalignments to genomic space using a model of transcriptome consisting of genes, transcripts and exon coordinates, which allows the interchange between pseudoalignment and genome alignment possible. We then evaluated the aligners on the count matrix output (Figure 1).

Specifically, we first checked the overall correlation of alignments from STAR and Kallisto workflows. We added a pseudo-count of 1 to all gene counts before log transformation, then calculated the Pearson’s correlation of all genes across all cells between STAR and Kallisto. As shown in Figure 1A-B, the correlation coefficient between Kallisto and STAR aligned gene counts is 0.836 and 0.862 for Drop-seq and Fluidigm data, respectively, demonstrating a strong concordance between them. However, further examination shows that STAR yields more uniquely expressed genes for both Drop-seq and Fluidigm platforms (Figure 1C-D). For Drop-seq data, STAR and Kallisto detect 16892 common genes, but 7116 and 1906 unique genes, respectively. For Fluidigm data, STAR and Kallisto detect 23193 common genes, but 13710 and 645 unique genes, respectively. The modes of the density distribution of gene numbers in each cell (Figure 1E-F) shift to higher values for STAR alignment, confirming that indeed STAR systematically detects more genes in each cell. Interestingly, while the Drop-seq platform yields a single peak of density distribution for gene counts, Fluidigm yields two peaks, both of which have higher gene counts than the peak of Drop-seq. Overall Kallisto pseudoaligned to more genes (proportion-wise) with shorter length (<3000bp), whereas STAR can handle longer gene alignment better, as shown in Figure 1G-H. Since dropout is a significant issue in single cell RNA-Seq data, we also compared dropout rates among commonly detected genes using cumulative density plots (Figure S1A-B). Overall, genes detected using STAR have cumulatively significantly lower dropout rates for both Drop-seq (K-S test p-value< 2.2 e-16) and Fluidigm (K-S test p-value < 2.2e-16) platforms.

In conclusion, despite high correlations between STAR and Kallisto, STAR detected more genes and also yielded more abundant gene expression counts across cells compared to Kallisto. However, since the absolute truth of gene expression is not used, the observations herein are relative measures.

### Validation of STAR and Kallisto results using RNA FISH data

**Figure 2:**
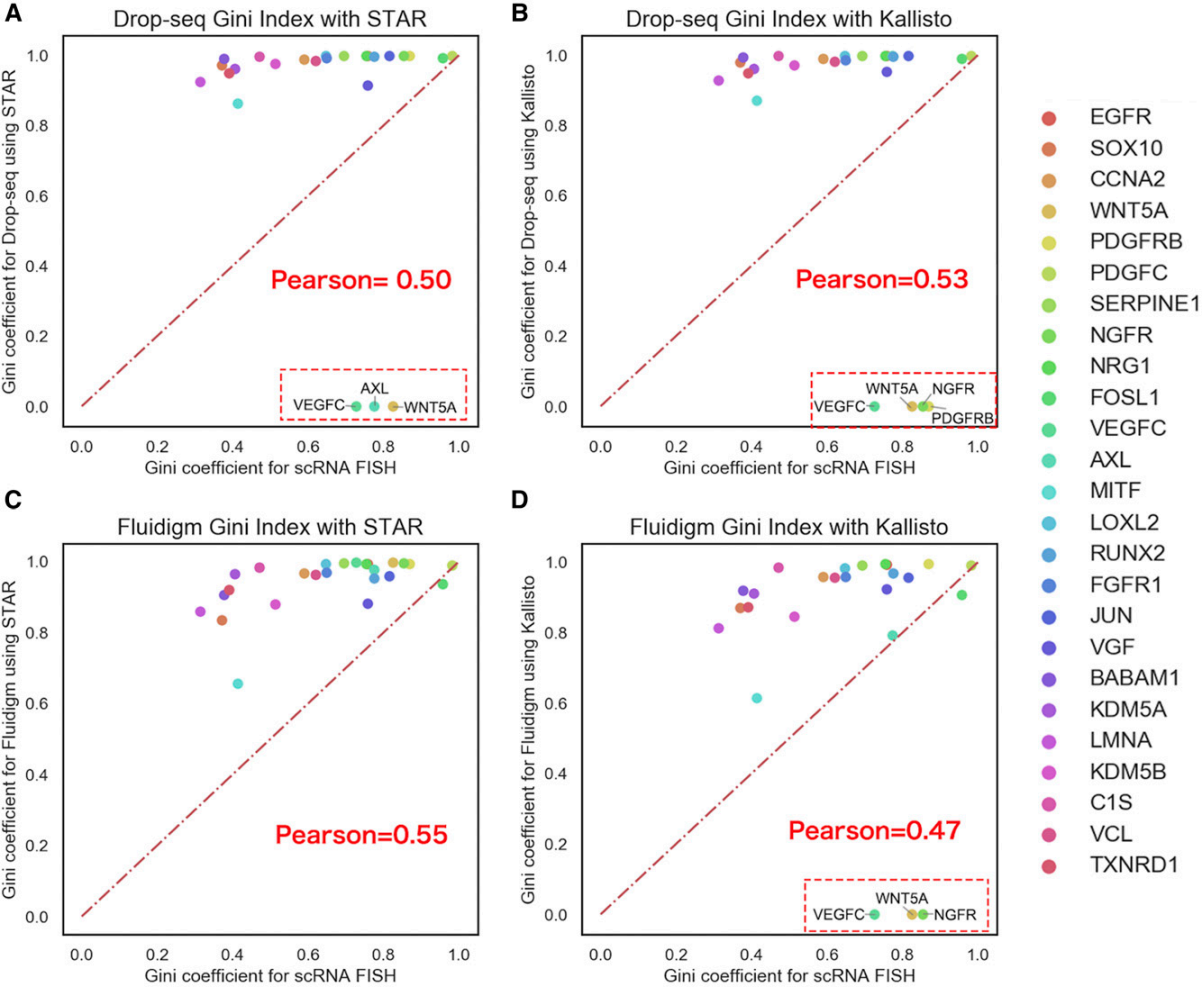
Validation of STAR and Kallisto results using RNA FISH data. (A-B) Scatterplots of Gini coefficients for Drop-seq *vs.* FISH data with STAR and Kallisto protocols for selected genes. The x-axis represents the Gini coefficient obtained from RNA FISH study, and the y-axis represents the Gini coefficient from the Drop-seq experiment using STAR and Kallisto respectively. (C-D) Scatterplots of Gini coefficients for Fluidigm *vs.* FISH data with STAR and Kallisto protocols. Pearson correlation coefficients are shown as Pearson (after removing the non-detected genes).

To address the issue of lack of absolute truth of gene expression in the comparisons above, we next assessed STAR and Kallisto performance using smRNA-FISH data as the ground truth measurement. Single-molecule RNA fluorescence *in situ* hybridization (smRNA FISH) is a method to detect RNA within a cell using the fluorescence probe. Torre *et al.* originally used smRNA-FISH to measure 26 genes and represented their distribution across cells using the Gini coefficient (Torre *et al.* 2018). Gini coefficient is a metric for inequality. A Gini coefficient of 0 represents that gene expression is perfectly even across all cells, whereas a Gini coefficient of 1 means that the gene is expressed in only one cell. In the context of gene expression, it can measure the variation of gene expression for a gene across all cells, and it is indeed influenced by the level of expression (see Equation 1). By comparing the Gini coefficient calculated from two alignment methods with that of the RNA Fish experiment, we can evaluate whether the alignment methods preserve the variability similar to the RNA Fish method. We compared the Gini coefficients between aligned scRNA-Seq data and smRNA-FISH data (Figure 2), and observed significant zero inflation in Gini coefficients from scRNA-Seq platforms, comparing to smRNA-FISH measurement (Torre *et al.* (Torre *et al.* 2018)). For Drop-seq data, as shown in Figure 2A-B, both STAR and Kallisto missed detecting some of the 26 genes. STAR missed three genes: VEGFC, AXL and WNT5A, whereas Kallisto missed four genes: VEGFC, WNT5A, NGFR, PDGFRB. After removing these outliers, the correlation between STAR Gini and smRNA-FISH Gini was 0.50, and the correlation between Kallisto Gini and smRNA-FISH Gini was 0.53. For Fluidigm data, as shown in Figure 2C-D, STAR detected all 26 genes, whereas Kallisto missed to detect 3 genes: VEGFC, WNT5A, and NGFR. The correlation between STAR Gini and smRNA-FISH Gini is 0.55, whereas the correlation between Kallisto Gini and smRNA-FISH was 0.47 (after removing undetected genes). In summary, the comparisons with smRNA-FISH data show that STAR tends to miss fewer genes. Moreover, STAR has on par with (Drop-seq) or slightly better (Fluidigm) correlations with smRNA-FISH based truth measure, despite the fact that the Gini coefficients between STAR and Kallisto are highly correlated for commonly detected genes (Figure S2A-B).

### Comparison of Bowtie2 *vs.* STAR and Kallisto alignment

We further compared Bowtie2, a popular alignment method on bulk RNA-Seq data, to STAR and Kallisto, on the above mentioned single cell datasets (Figure S3). Among all three methods, STAR still has the most abundant genes detected (24008). Bowtie2 is the second (21572), and Kallisto is the third (18789). However, Bowtie2 has slightly lower detected gene count per cell compared to both STAR and Kallisto (Figure S3 G and H). This indicates that even though Bowtie2 detected more unique gene counts than Kallisto, the average gene expression level across cells was lower than Kallisto. For Gini coefficient comparison, Bowtie2 has closer coordination with STAR (Figure S3 F), compared to Kallisto (Figure S3 E). STAR failed to detect two of 26 genes, whereas Bowtie2 and Kallisto both missed four genes (Figure S3 D-F). For efficiency, with premade reference, STAR took 15 min and 28G memory to finish the alignment, whereas Bowtie2 used 56 min and 14.2G memory. Therefore, by comparing with another traditional aligner Bowtie2, we have reached the consistent conclusion with other aligner benchmarking studies on the real and simulated datasets (Baruzzo *et al.* 2017); (Teissandier *et al.* 2019) that STAR is currently one of the top performing methods on alignment rates and speed comparing to other traditional aligners.

### Running time and memory comparison on Fluidigm data

**Figure 3:**
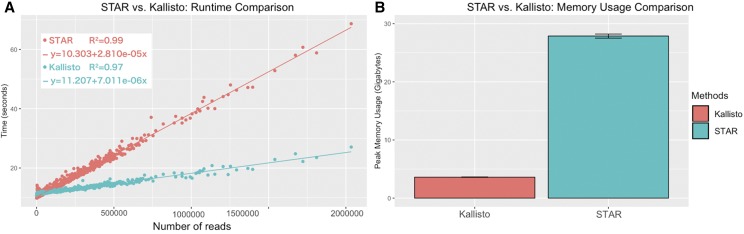
Time and memory comparison on Fluidigm datasets. (A) Computing time of Fluidigm data (800 cells) on the computer cluster (configurations in Methods section), using STAR and Kallisto. The x-axis represents the number of reads (in a given cell), and the y-axis represents the time usage in seconds. (B) Memory usage plot for Fluidigm data using STAR and Kallisto. The x-axis is the method, and the y-axis represents the memory usage in Gigabytes.

We performed time and memory usage comparison on a computer cluster (detailed configuration could be found in the Methods section) with Slurm job scheduler using Fluidigm data (800 cells). For Fluidigm data, the experiments were conducted using the same pipelines as in Figures 1 and 2, with the notation that STAR was aligned to reference genome GRCh38 whereas Kallisto used GRCh38 cDNA^+^intron index. Both Kallisto and STAR were assigned with one processor and 60GB memory and ran in one thread mode to minimize the effect of parallel processing. Overall, Kallisto pseudoalignment takes 1/4 amount of time as STAR (Figure 3A). Moreover, the maximum memory usage of Kallisto was ∼3.6GB, which is about 1/7 of the memory usage (∼28GB) of STAR (Figure 3B). Thus, Kallisto with cDNA-only index demonstrates significant advantages of pseudoalignment both on speed and memory.

### STARsolo and Kallisto bus comparison on human 10x Genomics PBMC data

In order to see the effect of align/pseudoalignment method on downstream clustering analysis, we compared STARsolo (Dobin *et al.* 2013) and Kallisto bustools (Melsted *et al.* 2019 *preprint*) on 10x genomics’ publically available PBMC 3K scRNA-Seq datasets. STARsolo (available in STAR after version 2.7.0) and Kallisto bustools are pipelines developed based on each method to analyze the UMI-based data, such as 10x genomics data. For fairness, cDNAs plus introns sequences were used as the reference to generate index files for both methods. Both pipelines produced the same output format from 10x’s Cell Ranger tool, so we next used the Seurat package (Butler *et al.* 2018; Stuart *et al.* 2019) to find cell clusters using unsupervised clustering method.

**Figure 4:**
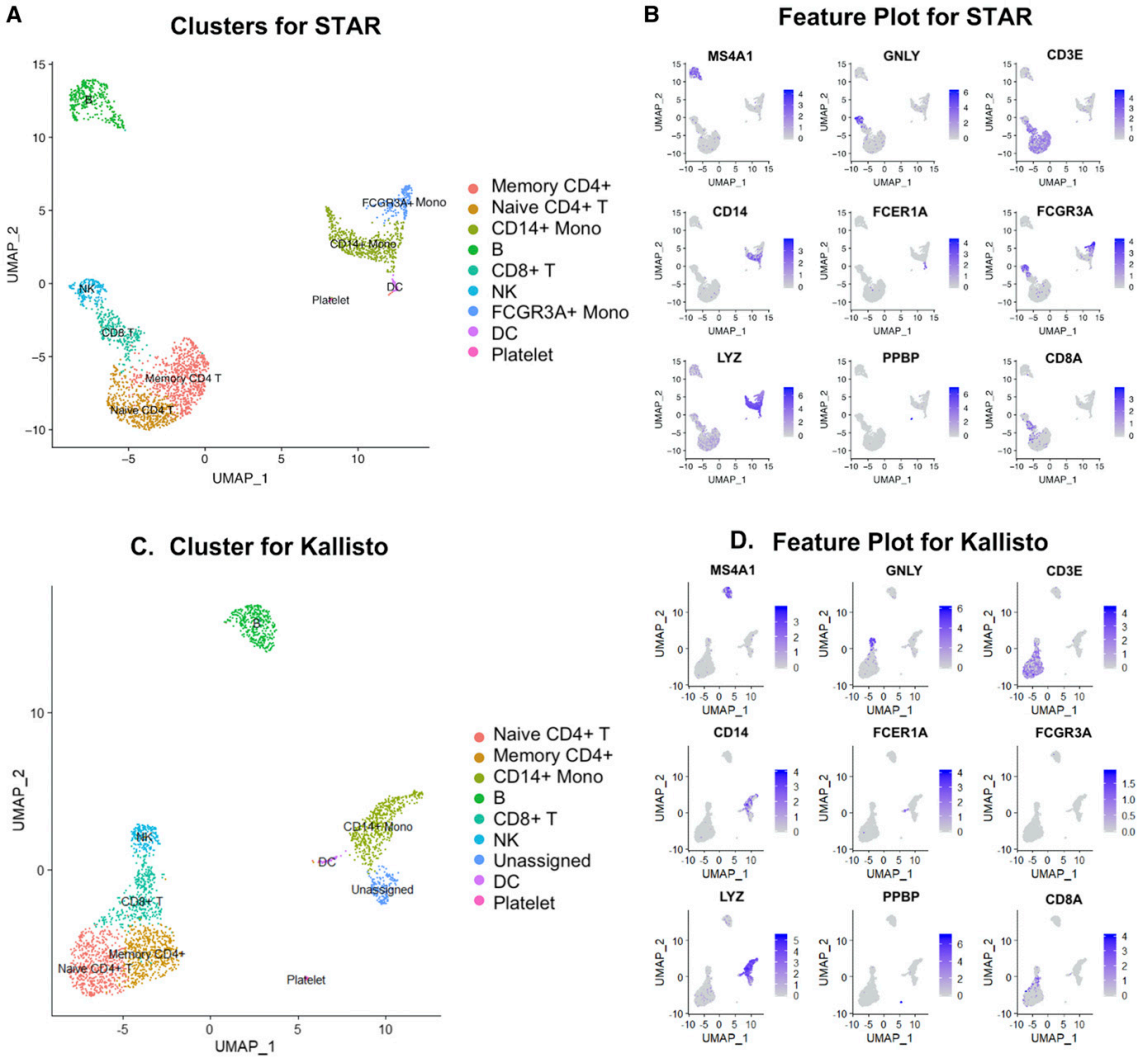
STARsolo *vs.* Kallisto result comparison on marker genes of 10x PBMC 3K data. (A, C) Clustering annotation using known cell-type specific markers, from results generated by STARsolo (A) and Kallisto with cDNA^+^intron index (C). The annotated cell types are: memory CD4 T cells (Memory CD4 T), naive CD4+ T cells (Naive CD4 T), CD14+ monocytes (CD14 + Mono), B cells (B), CD8+ T cells (CD8 T), Natural Killer cells (NK), FCGR3A+ monocytes (FCGR3A + Mono), Dendritic cells (DC), and platelet cells (Platelet). FCGR3A + Mono cell type cannot be assigned from results based on Kallisto. (B, D) Feature plot of known marker genes from results generated by STARsolo (B) and Kallisto (D).

For the PBMC 3K dataset, STARsolo showed 89.5% alignment rate, whereas Kallisto bustools has 61.89% pseudoalignment rate (Table S1). STARsolo output resulted in nine clusters (Figure 4A), each assigned to a cell type using the predefined markers in the Seurat vignette. 90.73% of cell labels from STARsolo matched with those from Cell Ranger (based on STAR), confirming the similarity between the two workflows (Table S2). Given the same reference genome (GRCh38), the difference between Cell Ranger and STARsolo could be caused by different correction and filtering criteria. In comparison, 1 out of 9 cell types, FCGR4A+Mono cells, could not be identified after Kallisto alignment (Figure 4C). This is largely due to the fact that Kallisto could not detect high enough FCER1A expression, one of the marker genes to identify the FCGR4A+Mono cell type (Figure 4D). For the proportions of cell types, STARsolo and Kallisto generate similar proportions for different cell types (Figure S4A), with STARsolo identifying more memory CD4+ T cells but fewer CD8+ T cells.

Worth noticing, the mapping rate (61.89%) using cDNA and intron index in Kallisto is much better than the mapping rate (51.30%) when only cDNA index was used. However, this improvement comes with the price of memory usage. With only cDNA index, Kallisto used 3.7 Gigabytes of memory, and the runtime was 46 min. With cDNA^+^intron index, Kallisto used 67.1 Gigabytes of memory, and the runtime was 1.5 hr. The clustering result from the Kallisto pipeline (with cDNA and intron index) is also much improved from that built on cDNA index. In the latter case, seven clusters are identified (Figure S5A), and the cell-type specific marker genes are either missing or do not co-locate well with the clusters (Figure S5B). Therefore, including introns in the reference genome can largely increase the accuracy for Kallisto.

### STARsolo and Kallisto bus comparison on single nuclei RNA-seq mouse cortex data

Single nuclei RNA-Seq is one solution for human studies where fresh materials are lacking (Lake *et al.* 2017). We downloaded the single nuclei Mouse Cortex 1 data published earlier (Ding *et al.* 2019 *preprint*). As single nuclei RNA-Seq data contain high content of intronic fragments, we evaluated the performance between STARsolo and Kallisto bus using cDNA^+^intron as the reference.

**Figure 5:**
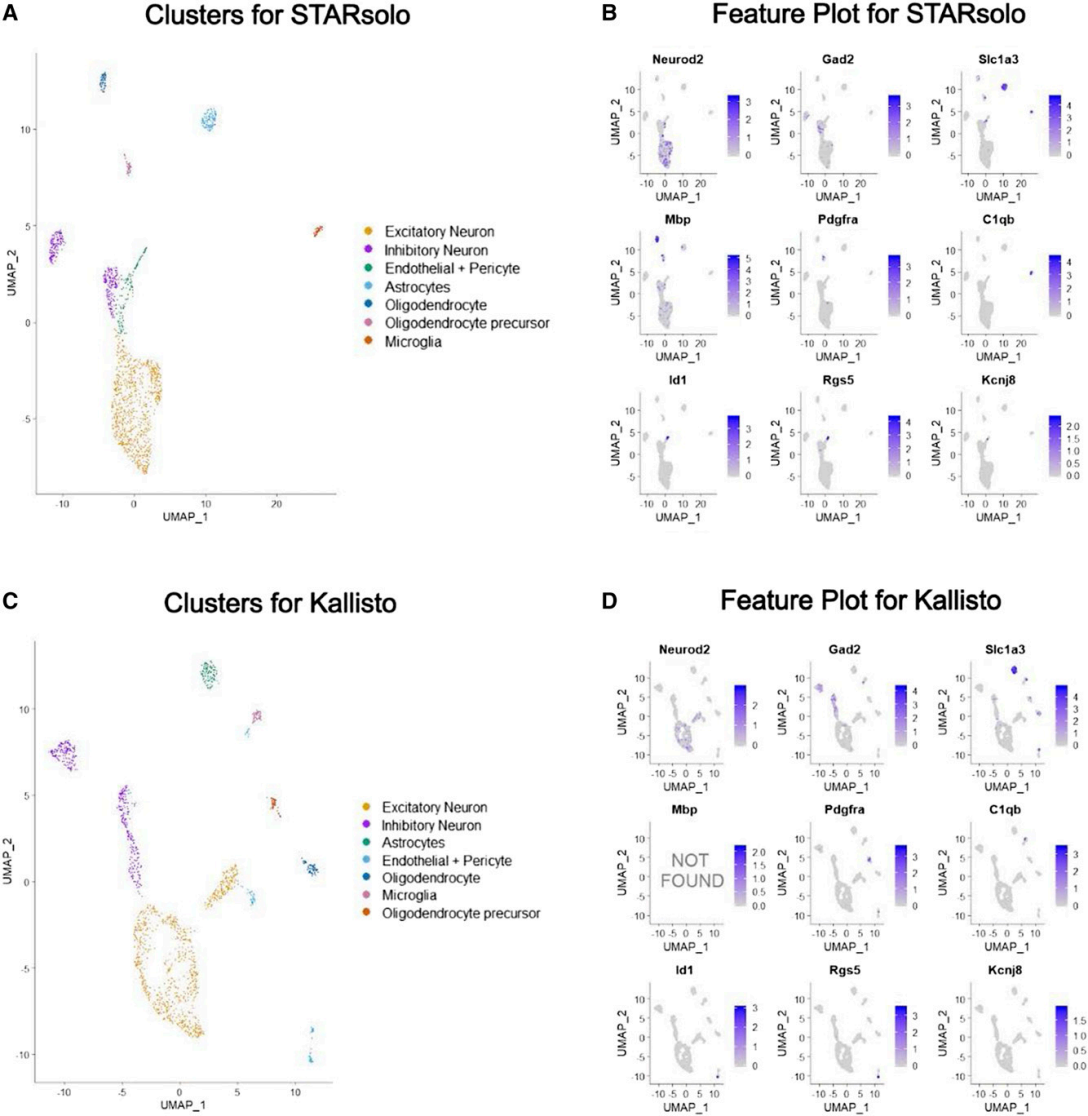
STARsolo *vs.* Kallisto on 10x Single nuclei Mouse Cortex (A, C) Clustering annotation using known cell-type specific markers, from results generated by STARsolo (A) and Kallisto with cDNA and intron index (C). Seven clusters are annotated as Excitatory Neuron,. Inhibitory Neuron, Endothelial + Pericyte, Astrocytes, Oligodendrocyte, Oligodendrocyte precursor, and Microglia. (B, D) Feature plot of known marker genes from results generated by STARsolo (B) and Kallisto (D). For Kallisto generated results, gene mbp was not detected across all cells hence shown as NOT FOUND.

For the mouse cortex single nuclei RNA-seq data, Kallisto bus required 58.9 Gigabytes of memory, whereas STARsolo used 31.4 Gigabytes. The run time was similar. Both STARsolo and Kallisto bus took 3 hr to complete (pseudo)alignment without the indexing step. STARsolo recovered 81.6% of reads, compared to 74.9% mapping rate for Kallisto (Table S1). This resulted in more genes detected by STARsolo. Both alignment methods detected seven cell types: excitatory neurons, inhibitory neurons, astrocytes, oligodendrocytes, oligodendrocyte precursors and microglia (Figure 5A and 5C). The proportions of detected cell types were similar using both STARsolo and Kallisto (Figure S4B). However, closer investigation showed that STARsolo detects more marker genes than Kallisto. For example, mbp, a marker gene for oligodendrocytes, is missing in Kallisto aligned result (Figure 5D *vs.* 5B). In fact, among the 47 cell-type specific markers, STARsolo only failed to identify one marker (Des), whereas Kallisto could not identify 7 markers (Table S3). In all, comparison on the single nuclei RNA-seq data further shows the significance to include intronic information for Kallisto alignment.

## Discussion

As single cell RNA-Seq (scRNA-Seq) technologies continue to gain popularity, the amount of generated data are increasing faster than ever, which subsequently imposes increasing demand to process these datasets. Alignment is one of the most time consuming yet most critical steps to process scRNA-Seq data. Currently, an unbiased third-party comparison on the alignment tools of scRNA-Seq data are lacking, although during the manuscript preparation time the authors of Kallisto had made a comparison preprint available (Melsted *et al.* 2019 *preprint*).

In this report, we compared the performance of the two most popular alignment/pseudoalignment methods: STAR and Kallisto. We aimed to provide readers the details on gene expression characteristics, beyond simple correlations between the two methods. Toward this goal, we purposefully selected datasets that have the “reference measures”. We first used a dataset with both scRNA-Seq (in both Drop-seq and Fluidigm platforms) and smRNA FISH validation results. Through the comparison, it appeared that in this dataset, despite high correlations between STAR and Kallisto, STAR tends to yield more genes overall, as well as more abundance of the genes, compared to Kallisto; whereas Kallisto detects more shorter genes compared to STAR. Based on the 26 genes that have smRNA FISH results, STAR appeared to detect more of them, compared to Kallisto; also STAR had on-par (Drop-seq) or slightly better (Fluidigm) correlations with the “reference measure” of smRNA-FISH.

We subsequently compared the alignment results using PBMC 3K cells, by examining the clustering results based on the count matrix. We chose this PBMC 3K dataset because the Seurat group provided marker genes and cell types, so we could use such knowledge as the “reference”. The result showed that STAR alignment harvested cell clusters that could be well identified by predefined cell-specific markers; the cell clusters generated from Kallisto with cDNA plus intron (not just cDNA) index information were very similar, but still missed one cell type. This dataset was not one of the 20 datasets reported by Melsted *et al.* (Melsted *et al.* 2019 *preprint*). We additionally applied these two methods on a single nuclei RNA-seq dataset from mouse cortex, in order to further investigate the necessity of including intronic reference for Kallisto. With cDNA and intron as the reference, Kallisto identifies the same cell types as STARsolo, despite missing slightly more marker genes. Thus, adding introns to cDNA sequences is vital to the alignment/ pseudoalignment step for Kallisto. Our results are consistent with Vieth *et al.* in their single cell pipeline evaluation studie: Kallisto with cDNA index had a low fraction of assigned reads, and for UMI-based methods STAR performs better (Vieth *et al.* 2019).

Consistent with the observation of Melsted *et al.*, we also found that Kallisto (with only cDNAs as the reference) is 4 times faster than STARsolo, the recent version of STAR aligner adapter for scRNA-Seq, and the memory usage of Kallisto is 7.7 times less than STARsolo. Including intron sequences in reference significantly improves the accuracy of Kallisto, however, this improvement is at the cost of computer memory. In summary, based on the datasets used in this study, we conclude that Kallisto’s use of computing resources is much less demanding than STAR when only cDNA sequences are used as the reference; however, such efficiency gain is at the cost of loss of information. The users should make decisions based on their preferences in accuracy *vs.* computing resources.

## References

[bib1] Arisdakessian C., Poirion O., Yunits B., Zhu X., and Garmire L. X., 2019 DeepImpute: an accurate, fast, and scalable deep neural network method to impute single-cell RNA-Seq data. Genome Biol. 20: 211 10.1186/s13059-019-1837-6 31627739 PMC6798445

[bib2] Baruzzo G., Hayer K. E., Kim E. J., Di Camillo B., FitzGerald G. A., 2017 Simulation-based comprehensive benchmarking of RNA-seq aligners. Nat. Methods 14: 135–139. 10.1038/nmeth.4106 27941783 PMC5792058

[bib3] Bray N. L., Pimentel H., Melsted P., and Pachter L., 2016 Near-optimal probabilistic RNA-seq quantification. Nat. Biotechnol. 34: 525–527. 10.1038/nbt.3519 27043002

[bib4] Butler A., Hoffman P., Smibert P., Papalexi E., and Satija R., 2018 Integrating single-cell transcriptomic data across different conditions, technologies, and species. Nat. Biotechnol. 36: 411–420. 10.1038/nbt.4096 29608179 PMC6700744

[bib5] Ding J., Adiconis X., Simmons S. K., Kowalczyk M. S., Hession C. C., 2019 Systematic comparative analysis of single cell RNA-sequencing methods. bioRxiv, (Preprint posted May 9, 2019). 10.1101/632216

[bib6] Dobin A., Davis C. A., Schlesinger F., Drenkow J., Zaleski C., 2013 STAR: ultrafast universal RNA-seq aligner. Bioinformatics 29: 15–21. 10.1093/bioinformatics/bts635 23104886 PMC3530905

[bib7] Engström P. G., Steijger T., Sipos B., Grant G. R., Kahles A., 2013 Systematic evaluation of spliced alignment programs for RNA-seq data. Nat. Methods 10: 1185–1191. 10.1038/nmeth.2722 24185836 PMC4018468

[bib8] Huang Q., Liu Y., Du Y., and Garmire L. X., 2019 Evaluation of Cell Type Deconvolution R Packages on Single Cell RNA-seq Data. bioRxiv, (Preprint posted November 1, 2019). 10.1101/827139

[bib9] Huang M., Wang J., Torre E., Dueck H., Shaffer S., 2018 SAVER: Gene expression recovery for single-cell RNA sequencing. Nat. Methods 15: 539–542. 10.1038/s41592-018-0033-z 29941873 PMC6030502

[bib10] Lake B. B., Codeluppi S., Yung Y. C., Gao D., Chun J., 2017 A comparative strategy for single-nucleus and single-cell transcriptomes confirms accuracy in predicted cell-type expression from nuclear RNA. Sci. Rep. 7: 6031 10.1038/s41598-017-04426-w 28729663 PMC5519641

[bib11] Langmead B., and Salzberg S. L., 2012 Fast gapped-read alignment with Bowtie 2. Nat. Methods 9: 357–359. 10.1038/nmeth.1923 22388286 PMC3322381

[bib12] Liao Y., Smyth G. K., and Shi W., 2014 featureCounts: an efficient general purpose program for assigning sequence reads to genomic features. Bioinformatics 30: 923–930. 10.1093/bioinformatics/btt656 24227677

[bib13] Liao Y., Smyth G. K., and Shi W., 2013 The Subread aligner: fast, accurate and scalable read mapping by seed-and-vote. Nucleic Acids Res. 41: e108 10.1093/nar/gkt214 23558742 PMC3664803

[bib14] Macosko E. Z., Basu A., Satija R., Nemesh J., Shekhar K., 2015 Highly Parallel Genome-wide Expression Profiling of Individual Cells Using Nanoliter Droplets. Cell 161: 1202–1214. 10.1016/j.cell.2015.05.002 26000488 PMC4481139

[bib15] Melsted P., Sina Booeshaghi A., Gao F., Beltrame E., Lu L., 2019 Modular and efficient pre-processing of single-cell RNA-seq. bioRxiv, (Preprint posted June 17, 2019). 10.1101/673285

[bib16] Ortega M. A., Poirion O., Zhu X., Huang S., Wolfgruber T. K., 2017 Using single-cell multiple omics approaches to resolve tumor heterogeneity. Clin. Transl. Med. 6: 46 10.1186/s40169-017-0177-y 29285690 PMC5746494

[bib17] Poirion O., Zhu X., Ching T., and Garmire L. X., 2018 Using single nucleotide variations in single-cell RNA-seq to identify subpopulations and genotype-phenotype linkage. Nat. Commun. 9: 4892 10.1038/s41467-018-07170-5 30459309 PMC6244222

[bib18] Stuart T., Butler A., Hoffman P., Hafemeister C., Papalexi E., 2019 Comprehensive Integration of Single-Cell Data. Cell 177: 1888–1902.e21. 10.1016/j.cell.2019.05.031 31178118 PMC6687398

[bib19] Teissandier A., Servant N., Barillot E., and Bourc’his D., 2019 Tools and best practices for retrotransposon analysis using high-throughput sequencing data. Mob. DNA 10: 52 10.1186/s13100-019-0192-1 31890048 PMC6935493

[bib20] Torre E., Dueck H., Shaffer S., Gospocic J., Gupte R., 2018 Rare cell detection by single cell RNA sequencing as guided by single molecule RNA FISH. Cell Syst. 6: 171–179.e5. 10.1016/j.cels.2018.01.014 29454938 PMC6078200

[bib21] Vieth B., Parekh S., Ziegenhain C., Enard W., and Hellmann I., 2019 A systematic evaluation of single cell RNA-seq analysis pipelines. Nat. Commun. 10: 4667 10.1038/s41467-019-12266-7 31604912 PMC6789098

[bib22] Yang C., Wu P.-Y., Tong L., Phan J. H., and Wang M. D., 2015 The impact of RNA-seq aligners on gene expression estimation. ACM BCB 2015: 462–471. 27583310 10.1145/2808719.2808767 PMC5003035

[bib23] Yi L., Liu L., Melsted P., and Pachter L., 2018 A direct comparison of genome alignment and transcriptome pseudoalignment. bioRxiv, (Preprint posted October 16, 2019). 10.1101/444620

[bib24] Zhang C., Zhang B., Lin L.-L., and Zhao S., 2017 Evaluation and comparison of computational tools for RNA-seq isoform quantification. BMC Genomics 18: 583. 28784092 10.1186/s12864-017-4002-1 PMC5547501

[bib25] Zhu X., Wolfgruber T. K., Tasato A., Arisdakessian C., Garmire D. G., 2017 Granatum: a graphical single-cell RNA-Seq analysis pipeline for genomics scientists. Genome Med. 9: 108 10.1186/s13073-017-0492-3 29202807 PMC5716224

[bib26] Zhu X., Yunits B., Wolfgruber T., Liu Y., Huang Q., 2019 GranatumX: A community engaging and flexible software environment for single-cell analysis. bioRxiv, (Preprint posted August 6, 2018). 10.1101/385591

